# A dual-genotype oligoastrocytoma with histologic, molecular, radiological and time-course features

**DOI:** 10.1186/s40478-020-00998-3

**Published:** 2020-07-20

**Authors:** Mac Lean P. Nasrallah, Arati Desai, Donald M. O’Rourke, Lea F. Surrey, Joel M. Stein

**Affiliations:** 1 Division of Neuropathology, Department of Pathology and Laboratory Medicine, Perelman School of Medicine at the University of Pennsylvania, Philadelphia, PA 19104 USA; 2 Department of Medicine, Perelman School of Medicine at the University of Pennsylvania, Philadelphia, PA 19104 USA; 3 Department of Neurosurgery, Perelman School of Medicine at the University of Pennsylvania, Philadelphia, PA 19104 USA; 4 Department of Pathology and Laboratory Medicine, Children’s Hospital of Philadelphia, Philadelphia, PA 19104 USA; 5 Division of Neuroradiology, Department of Radiology, Perelman School of Medicine at the University of Pennsylvania, Philadelphia, PA 19104 USA

**Keywords:** Oligoastrocytoma, IDH, 1p/19q, T2-FLAIR mismatch

## Abstract

A case of a true dual-genotype *IDH*-mutant oligoastrocytoma with two different cell types within a single mass in a young woman is presented. Imaging findings of the left frontal infiltrating glioma predicted the two neoplastic components that were identified upon resection. Tissue examination demonstrated areas of tumor with contrasting histologic and molecular features, including specific *IDH1*, *ATRX*, *TP53*, *TERT* and *CIC* mutational profiles, consistent with oligodendroglioma and astrocytoma, respectively. The clinical and radiological course over 17 months from first diagnosis included three surgical resections with slow progression of the astrocytic component, and ultimately chemotherapy and radiation treatments were commenced. Reports of the clinical courses for these rare cases of dual-genotype oligoastrocytomas will inform therapy choices, to optimize benefit while minimizing side effects. The steadily increasing number of cases suggests that the neoplasm might be reconsidered as an official entity by the WHO.

## Introduction

Historically, oligoastrocytoma has been defined as an infiltrating glioma composed of two distinct neoplastic cell types, with oligodendroglial and astrocytic features, respectively [8]. According to the 2016 CNS WHO, the diagnosis of oligoastrocytoma is exceptional and essentially should not be made given the ability of molecular testing to differentiate between the two types of glioma [9]. Although “oligoastrocytoma, dual-genotype” is described in the 2016 CNS WHO, it is not considered a distinct entity or variant of glioma. Characterization of *ATRX* and *TP53* variants along with 1p/19q codeletion testing results allow almost all cases of *IDH*-mutant gliomas to be diagnosed unambiguously as oligodendrogliomas or astrocytomas, with *TERT* promoter, *CIC* and *FUBP1* variants adding further clarity [4, 7, 15, 18]. Molecular genetic definitions have also facilitated analysis or reanalysis of the radiological features of these tumor subtypes. For example, genetic oligodendrogliomas tend to show indistinct borders and heterogenous signal intensity on T1- and T2-weighted images [6]. Astrocytomas appear more circumscribed and homogeneous on these sequences, but may demonstrate loss of central T2-hyperintense signal on FLAIR images or “T2-FLAIR mismatch”, a specific imaging marker of the IDH-mutant 1p19q non-codeleleted genotype [12]. Here we report a case in which pre-operative imaging suggested the presence of two separate tumor genotypes with extensive molecular characterization supporting the existence, albeit rare, of true oligoastrocytoma.

## Case presentation

We present the case of a 29-year-old female with a left frontal glioma, which demonstrates two components that are morphologically, immunohistologically, molecularly and radiologically distinguishable as “oligodendroglial” and “astrocytic.” The patient initially presented with a new onset generalized seizure, preceded by 2 weeks of increasing headache, restlessness and intermittent fatigue.

**Fig. 1:**
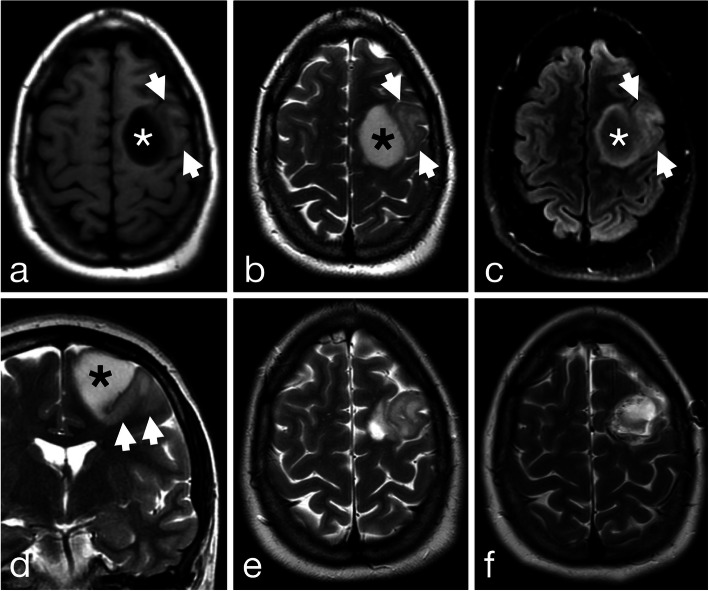
High left frontal tumor with two radiologically distinct components on preoperative axial T1-weighted (**a**), T2-weighted (**b**), FLAIR and coronal T2-weighted (**d**) MR images. A medial T1-hypointense T2-hyperintense component (asterisk) centered in the superior frontal gyrus shows well defined margins and the “T2-FLAIR” mismatch sign indicative of IDH1 mutant 1p/19q non-codeleted astrocytoma. A contiguous more lateral component (arrowheads) centered in the middle frontal gyrus shows less well-defined margins with cortical infiltration and gyral expansion more typical of oligodendroglioma. Follow-up axial T2-weighted imaging after resection of the more medial component (**e**) and the more lateral component (**f**)

Initial magnetic resonance imaging (MRI) demonstrated a high left frontal mass with two radiologically distinct components (Fig. 1a-d). A more medial component centered in the superior frontal gyrus consisted of a large circumscribed rounded T1-hypointense and T2-hyperintense area with central signal suppression on FLAIR images (the “T2-FLAIR mismatch sign”) indicative of IDH-1 mutant 1p/19q non-codeleted astrocytoma. A contiguous more lateral component within the middle frontal gyrus showed a different pattern with more heterogeneous signal intensity, ill-defined margins, and cortical infiltration with gyral expansion more typical of oligodendroglial tumors. There was no evidence of calcification as can be seen with oligodendroglioma on MRI or computed tomography (CT). There was no associated enhancement and no elevated perfusion or permeability parameters on dynamic susceptibility contrast or dynamic contrast enhanced imaging in either region. The patient underwent a partial resection with removal of the more medial component (Fig. 1e). Additional surgery was performed 7 months later with gross total resection of the more lateral component (Fig. 1f).

**Fig. 2:**
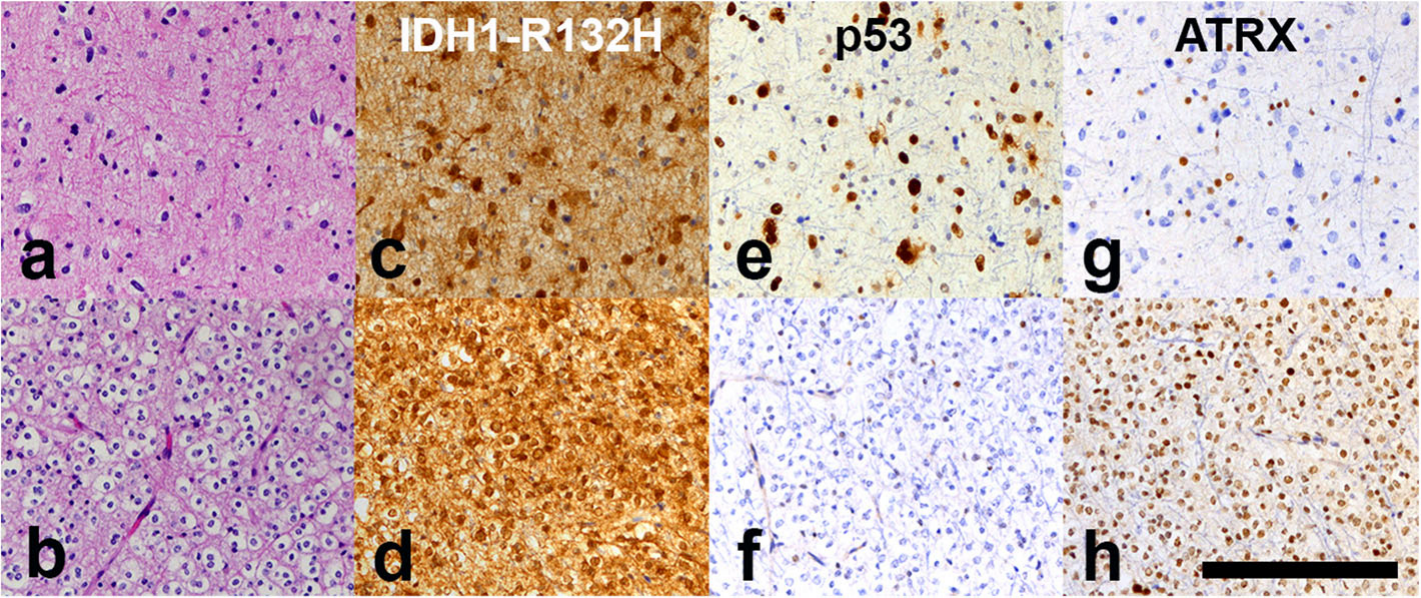
Astrocytic (**a**) and oligodendroglial (**b**) areas, both *IDH*-mutant (**c**, **d**) with contrasting p53 and ATRX staining patterns (**e**-**h**) (scale bar 200 μm)

Tissue examination of the resection of the medial component demonstrated low-grade IDH-mutant astrocytoma (Supplemental Fig. 1a-d). The patient received no further treatment at that time, but 7 months later, the patient and her care team chose to pursue further resection of the residual glioma, given the survival benefit of complete gross resection [11]. The subsequent gross total resection of the lateral component yielded histologic sections that show a cellular infiltrating glial tumor composed of two morphologic patterns. In some areas, the tumor cells have irregular, elongated nuclei with dense chromatin, more consistent with an infiltrating astrocytoma (Fig. 2a). In other areas, perinuclear clearing of the cytoplasm and round nuclei with open chromatin are observed, histologically consistent with an oligodendroglioma (Fig. 2b). In the latter areas, a network of fine capillary blood vessels is noted. No significant mitotic activity, necrosis or microvascular proliferation is seen in any area. Although the two morphologies are largely geographically separate, areas are seen where the two morphologies appear mutually infiltrative, although immunohistochemical stains failed to clearly demonstrate the intermingling of the two glioma types.

Contrasting patterns of positivity for immunohistochemical stains for GFAP, mutant IDH1-R132H, p53 and ATRX underscore and augment the difference between the two cell populations (Fig. 2c-h). Although all tumor cells are positive for GFAP and IDH1-R132H, the astrocytic population show strong nuclear positivity for p53 and loss of nuclear ATRX expression (Fig. 2e,g), whereas the oligodendroglial population shows minimal p53 staining with retention of ATRX nuclear expression (Fig. 2f,h). In addition, fluorescent in situ hybridization (FISH) studies for 1p/19q codeletion demonstrated codeletion in the oligodendroglial component, but not in the astrocytic component. MGMT promoter methylation testing by pyrosequencing of four CpG sites on an area of tumor that included both components gave a low positive result [10].

Next-generation sequencing (NGS) studies were performed as previously described [17] on the initial resection as well as on the two different components of the subsequent resection (Table 1, Supplemental Tables 1 and 2). The initial resection and subsequent astrocytic component demonstrated disease-associated variants in *IDH1*, *TP53*, and *ATRX*, whereas the subsequent oligodendroglial component showed the same variant in *IDH1* as well as variants in the *TERT* promoter and *CIC*. Copy number analysis of the NGS data demonstrated whole-arm deletion of 1p and 19q in the oligodendroglial portion only. No additional copy number alterations were detected in any sample. Inspection of the individual sequencing reads demonstrates minimal overlap of mutations with the exception of the variant of *IDH1* and *MDM4* p.Asp153Gly, which is of uncertain significance and may be of germline origin based on the higher variant allele fraction in both tumors and reports in population databases. These findings are consistent with the histologic findings and immunohistochemical results. Of note, CDKN2A/2B homozygous deletion was absent [2].

**Table 1:** Genomic alterations identified by NGS in each tumor component

Genomic Alteration			Variant Allele Fraction or % mosaicism		
Gene	DNA Change	Protein Change	Oligodendroglioma	Astrocytoma	Initial Resection
*IDH1*	c.395G > A (NM_005896.3)	p.Arg132His	0.31	0.29	0.43
*TERT*	c.-124C > T (NM_198253.2)	p.?	0.23	0	0
*CIC*	c.781_792del (NM_015125.4)	p.Phe261_His264del	0.32	0	0
*–*	1p/19q co-deletion	–	35–45%	not detected (< 30%)	not detected (< 30%)
*TP53*	c.841G > A (NM_000546.5)	p.Asp281Asn	< 0.01	0.38	0.87
*TP53*	c.817C > T (NM_000546.5)	p.Arg273Cys	< 0.01	0.08	0.08
*ATRX*	c.4744dup (NM_000489.4)	p.Thr1582Asnfs*19	< 0.01	0.26	0.26
*NOTCH1*	c.1347C > G (NM_017617.4)	p.Cys449Trp	0	0.18	0.18
*PIK3CA*	c.3197C > T (NM_006218.3)	p.Ala1066Val	0	0	0.05
*MDM4*	c.458A > G (NM_002393.4)	p.Asp153Gly	0.39	0.44	0.44

After her second resection, the patient returned to work and was followed by close clinical and imaging observation, without radiation or chemotherapy. On follow-up imaging, no progression was noted over the course of 4 months after the second resection, but slow progression was seen at 6 and 8 months after the second resection (13 and 15 months after initial presentation). At that time, the patient noted fatigue, as well as cognitive and speech decline. MRI demonstrated increasing T2/FLAIR nonenhancing signal abnormality at the margins of resection in the superior frontal gyrus and extending inferiorly to the corona radiata. Given the time interval of the follow up, the extent of the change was more than expected for a low grade glioma and in the absence of radiation therapy. A third surgery 10 months after the second resection demonstrated infiltrating low-grade glioma with a prominent astrocytic component (Supplemental Fig. 1e-h), and the patient subsequently received concurrent temozolomide with radiation followed by adjuvant temozolomide. The glioma was classified as a “high risk” lower grade glioma based on the presence of residual tumor [11]. Therefore, temozolomide was chosen as a chemotherapeutic agent over procarbazine, lomustine and vincristine (PCV) given the evidence of the efficacy of temozolomide in aggressive astrocytoma, the lack of clear evidence of the superiority of PCV over temozolomide in high risk astrocytoma [3], and the greater toxicity and side effects of PCV.

## Discussion and conclusions

Several other “dual-genotype oligoastrocytomas” have been reported [5, 19, 20]. Underscoring the rarity of this tumor type, studies with molecular profiling of 43 and 405 gliomas, respectively, allowed all of the gliomas to be definitively classified as oligodendrogliomas or astrocytomas, without necessitating a diagnosis of mixed oligoastrocytoma [14, 15]. However, for the latter and larger series, it is unclear whether morphologically heterogeneous areas within the gliomas were present and would have been analyzed. Our case underscores the continued importance of histological and immunohistochemical analysis in the current era of molecular testing, as well as correlation with radiological and surgical findings to avoid undersampling. Multiple areas of neoplasms are not routinely assessed by molecular analysis. If sequencing had been performed on a single minute fragment of tumor without awareness of the complete specimen’s diverse morphological and immunohistochemical characteristics, the dual genotype would not have been detected. In addition, the process is dependent on representative resected tissue being sent by the surgeon for pathologic analysis. Otherwise, for a dual-genotype oligoastrocytoma, even the complete battery of histological, immunohistochemical and molecular testing from a single sample is likely to lead to an unambiguous but incomplete diagnosis of either oligodendroglioma or astrocytoma without detection of a mixture.

In our case, inspection of the individual sequencing reads in the two areas of tumor demonstrated minimal overlap of associated mutations with the exception of the *IDH1* p.Arg132His. Similarly, Qu et al. found different molecular results in morphologically distinct areas of two out of 11 histological oligoastrocytomas, with no evidence of any of the 11 tumors showing the co-existence of loss of heterozygosity of 1p/19q and *TP53* variants in the same area of tumor even when cells with the different histological properties were diffusely admixed [13]. In contrast, Wilcox et al. found admixed oligodendroglial and astrocytic neoplastic cells in two tumors [19].

Ultimately, the question is how patients with dual oligoastrocytomas should be treated. In both our case and a case reported by Huse et al. [5], the astrocytic component recurred, although in our case, the neoplasm has not progressed to a higher grade, consistent with MGMT promoter methylation and lack of CDKN2A/2B homozygous deletion [1, 16]. Although each patient’s circumstances are unique and will dictate the choice of treatment, in the setting of clear residual disease, current practice in neuro-oncology may best consider these neoplasms as high-risk low-grade gliomas, even for a young patient. In that case, appropriate treatment would be adjuvant radiotherapy and chemotherapy based on RTOG 9802 [3].

In conclusions, histological/immunohistochemical analysis in the current era of molecular testing remains essential, and may be informed by radiologic analysis discriminating glioma types. Molecular oligoastrocytomas studied to date fall into one of two genetic categories. Either two subclones are present representing oligodendroglioma and astrocytoma, respectively, or one clone with a molecular profile that is a hybrid of the two types is seen [5, 19, 20]. Our case falls into the former class, with genetics suggesting the development of two divergent subclones from a common *IDH*-mutant predecessor, although a collision tumor is possible. In both situations, the prognosis, its drivers, and optimal treatment are uncertain. However, the clinical course of this patient with slow progression of tumor that was pathologically determined to consist predominantly of astrocytoma suggests that the astrocytic component dictates outcome.

## Supplementary information

**Additional file 1: Supplemental Fig. 1.** The initial resection of the medial component of the tumor demonstrated infiltrating glioma with both astrocytic and oligodendroglial histologic features (a). Immunohistochemical stains were performed on multiple blocks, and showed a consistent astrocytoma pattern, despite the oligodendroglial features. The glioma was positive for the IDH1 p.R132H variant (b). Strong, nuclear staining for p53 was present in a subset of tumor nuclei (c), and ATRX was absent in a majority of tumor nuclei, with positive staining in endothelial cell nuclei and small scattered nuclei (d). The third resection demonstrated scattered tumor cells at the edge of the prior resection cavity (e), with IDH (f) and p53 (g) positivity. Many macrophages and microglia are highlighted by CD68 (h) (scale bar 200 μm)

**Additional file 2.**

**Additional file 3.**

## Data Availability

All data generated or analysed during this study are included in this published article and its supplementary information files.

## Abbreviations

MRI: magnetic resonance imaging
NGS: Next-generation sequencing
