# Supplementary material for: A dual-genotype oligoastrocytoma with histologic, molecular, radiological and time-course features
Source: Acta Neuropathol Commun. 2020 Jul 20;8:115. doi: 10.1186/s40478-020-00998-3 (PMC7372861; doi:10.1186/s40478-020-00998-3)
Supplement: Supplementary file 1 — Additional file 1: Supplemental Fig. 1. The initial resection of the medial component of the tumor demonstrated infiltrating glioma with both astrocytic and oligodendroglial histologic features (a). Immunohistochemical stains were performed on multiple blocks, and showed a consistent astrocytoma pattern, despite the oligodendroglial features. The glioma was positive for the IDH1 p.R132H variant (b). Strong, nuclear staining for p53 was present in a subset of tumor nuclei (c), and ATRX was absent in a majority of tumor nuclei, with positive staining in endothelial cell nuclei and small scattered nuclei (d). The third resection demonstrated scattered tumor cells at the edge of the prior resection cavity (e), with IDH (f) and p53 (g) positivity. Many macrophages and microglia are highlighted by CD68 (h) (scale bar 200 μm) [file 40478_2020_998_MOESM1_ESM.docx]

A dual-genotype oligoastrocytoma with histologic, molecular, radiological and time-course features

MacLean P. Nasrallah^1^, Arati Desai^2^, Donald M. O’Rourke^3^, Lea F. Surrey^4^, Joel Stein^5^

Supplemental figure 1. The initial resection of the medial component of the tumor demonstrated infiltrating glioma with both astrocytic and oligodendroglial histologic features (a). Immunohistochemical stains were performed on multiple blocks, and showed a consistent astrocytoma pattern, despite the oligodendroglial features. The glioma was positive for the IDH1 p.R132H variant (b). Strong, nuclear staining for p53 was present in a subset of tumor nuclei (c), and ATRX was absent in a majority of tumor nuclei, with positive staining in endothelial cell nuclei and small scattered nuclei (d). The third resection demonstrated scattered tumor cells at the edge of the prior resection cavity (e), with IDH (f) and p53 (g) positivity. Many macrophages and microglia are highlighted by CD68 (h) (scale bar 200 microns)
